# Protein Identification of Spermatozoa and Seminal Plasma in Bottlenose Dolphin (*Tursiops truncatus*)

**DOI:** 10.3389/fcell.2021.673961

**Published:** 2021-07-16

**Authors:** Mari-Carmen Fuentes-Albero, Leopoldo González-Brusi, Paula Cots, Chiara Luongo, Silvia Abril-Sánchez, José Luis Ros-Santaella, Eliana Pintus, Sara Ruiz-Díaz, Carlos Barros-García, María-Jesús Sánchez-Calabuig, Daniel García-Párraga, Manuel Avilés, Mᵃ José Izquierdo Rico, Francisco Alberto García-Vázquez

**Affiliations:** ^1^Department of Biology, Avanqua-Oceanogràfic S.L, Valencia, Spain; ^2^Department of Physiology, Faculty of Veterinary Science, University of Murcia, Murcia, Spain; ^3^Department of Cell Biology and Histology, Faculty of Medicine, University of Murcia, Murcia, Spain; ^4^Department of Veterinary Sciences, Faculty of Agrobiology, Food and Natural Resources, Czech University of Life Sciences Prague, Prague, Czechia; ^5^Department of Animal Reproduction, National Agricultural and Food Research and Technology Institute (INIA), Madrid, Spain; ^6^Department of Medicine and Surgery, Faculty of Veterinary Science, Madrid, Spain; ^7^Research Department, Fundación Oceanogràfic, Valencia, Spain

**Keywords:** cetacean, proteomic analyses, semen, sperm cells, seminal plasma

## Abstract

Proteins play an important role in many reproductive functions such as sperm maturation, sperm transit in the female genital tract or sperm-oocyte interaction. However, in general, little information concerning reproductive features is available in the case of aquatic animals. The present study aims to characterize the proteome of both spermatozoa and seminal plasma of bottlenose dolphins (*Tursiops truncatus*) as a model organism for cetaceans. Ejaculate samples were obtained from two trained dolphins housed in an aquarium. Spermatozoa and seminal plasma were analyzed by means of proteomic analyses using an LC-MS/MS, and a list with the gene symbols corresponding to each protein was submitted to the DAVID database. Of the 419 proteins identified in spermatozoa and 303 in seminal plasma, 111 proteins were shared by both. Furthermore, 70 proteins were identified as involved in reproductive processes, 39 in spermatozoa, and 31 in seminal plasma. The five most abundant proteins were also identified in these samples: AKAP3, ODF2, TUBB, GSTM3, ROPN1 for spermatozoa and CST11, LTF, ALB, HSP90B1, PIGR for seminal plasma. In conclusion, this study provides the first characterization of the proteome in cetacean sperm and seminal plasma, opening the way to future research into new biomarkers, the analysis of conservation capacity or possible additional applications in the field of assisted reproductive technologies.

## Introduction

Cetaceans, aquatic mammals from the Cetacea infraorder, are considered essential for marine ecosystems (Bowen, 1997; Uhen, 2007). Their populations are under enormous anthropogenic pressure, especially as a result of commercial overfishing, incidental captures in fisheries, and habitat degradation (Reeves et al., 2003; Parsons et al., 2014; Dolman and Brakes, 2018). Indeed, many cetacean species are threatened or in danger of extinction (Davidson et al., 2012). This is why cetaceans are protected under many national, regional and international legislations including Appendix II of the Convention on International Trade in Endangered Species of Wild Fauna and Flora (CITES), which limits the movement of animals or their products between countries. However, study of their populations in the wild involves many limitations (Bowen, 1997; O’Brien and Robeck, 2010) and, in most cases, it is not possible to keep individuals in captivity due to their size, their gregarious population structure or other peculiarities of their ethology that make their *ex situ* maintenance highly difficult or almost impossible. Only some species, such as *Tursiops truncatus*, the common bottlenose dolphin, have a docile character and the ability to adapt well to captivity, which makes them ideal model organisms (Barratclough et al., 2019). Moreover, important information can be obtained from *ex situ* populations in order to deepen our knowledge of their features for conservation programs, but also to extrapolate the obtained knowledge to address problems related with other cetacean species (Barratclough et al., 2019).

During recent decades, aquariums and animal conservation centers have actively contributed to the conservation and reproduction of bottlenose dolphins through joint breeding programs (Katsumata, 2010). Initially, the movement and exchange of animals for reproductive purposes between centers was necessary, but this activity entailed certain risks that could affect the integrity of the animals (O’Brien and Robeck, 2010). Subsequently, the progress made in assisted reproductive technologies (ARTs) allowed the collection and management of gametes, thus ensuring their quality to maximize fertilization (Beirão et al., 2019). In order to minimize any impact on animal welfare, voluntary semen collection techniques through animal training have been routinely established in dolphin populations under human care (Robeck and O’Brien, 2004; Robeck et al., 2005). The collection of semen facilitates research activity and the exchange of reproductive cells between geographically distant centers for further use in artificial insemination programs (Robeck et al., 2005). For that purpose, methods based on refrigeration (Takenaka et al., 2013; Ruiz-Díaz et al., 2020) and freezing (Robeck and O’Brien, 2004; Robeck et al., 2013; Sánchez-Calabuig et al., 2015b, 2017) have been developed for dolphins, including techniques for preservation of seminal samples that have allowed the creation of a gene bank of high biological value for use in the future (Holt and Pickard, 1999).

A knowledge of the semen characteristics of a species of interest is essential to gain new insights, either to widen fundamental basic studies or for further application in ARTs. Semen is composed of the cellular part, the spermatozoa, and the liquid part, the seminal plasma. Bottlenose dolphin spermatozoa are known to have a long tail, a short but hydrodynamic head shape and a bulky midpiece (van der Horst et al., 2018), these high quality features being kept after collection (van der Horst et al., 2018; Ruiz-Díaz et al., 2020). Seminal plasma is a fluid coming mostly from the prostate, the only sexual accessory gland described in cetaceans (Harrison et al., 1969; Rommel et al., 2007; Suárez-Santana et al., 2020). The complex composition of seminal plasma plays a key role in both the male and female reproductive features of mammals. In the case of males, seminal plasma is involved in sperm maturation, motility, transport, capacitation or acrosome reaction (reviewed by Juyena and Stelletta, 2012). When the seminal plasma is deposited in the female genital tract, it affects the inflammatory and immune responses (reviewed by Bromfield, 2018), protecting sperm (Kawano et al., 2014; Luongo et al., 2019), and even having an impact on the offspring (Bromfield et al., 2014). Furthermore, it has been demonstrated that the proteins of reproductive fluids (including oviductal and uterine fluids, and seminal plasma) are involved in the interaction with the sperm proteome (Luongo et al., 2020; Rickard and de Graaf, 2020).

Proteomic analysis has been used to describe the sperm and seminal plasma proteome either in non-mammal species, such as fish (Ciereszko et al., 2017; Dietrich et al., 2017), and in mammals, such as murine (Baker et al., 2008; Vicens et al., 2017), porcine (Perez-Patiño et al., 2016; Recuero et al., 2019; Luongo et al., 2020), equine (Swegen et al., 2015; Guasti et al., 2020), ovine (Cardozo et al., 2006; Pini et al., 2016), caprine (Pinto et al., 2019; Zhu et al., 2020; Martínez-Fresneda et al., 2021), bovine (Peddinti et al., 2008; Byrne et al., 2012), non-human primates (Skerget et al., 2013), and human (Martínez-Heredia et al., 2006), but not yet in any cetacean species. Furthermore, such studies have permitted the identification of proteins that can act as biomarkers of fertility (Dacheux et al., 2012; Li et al., 2016; Rahman et al., 2017; Druart and de Graaf, 2018; Pérez-Patiño et al., 2018; Druart et al., 2019), their relevance for sperm preservation (Soleilhavoup et al., 2014; Parrilla et al., 2019; Peris-Frau et al., 2019; Ryu et al., 2019; Bajuk et al., 2020; De Lazari et al., 2020) or involvement in sperm functional traits (Intasqui et al., 2016; Bezerra et al., 2019; De Lazari et al., 2019). Moreover, seminal plasma proteins are influenced by the social and competitive environment (Ramm, 2014; Mead, 2018; Hopkins et al., 2019). Therefore, knowing the protein profile of semen dolphins may be especially relevant to better understand the biology of the species in light of the complex multi-male mating strategy of bottlenose dolphins.

Finally, knowing the proteome of ejaculated spermatozoa and seminal plasma may contribute to obtaining comprehensive information and to understanding functional implications for reproductive processes not only for dolphin species but for cetaceans in general. For this reason, this study aims to identify, describe, and classify for the first time, the sperm and seminal plasma proteins of dolphins and to compare them with the protein profiles in other species (bovine, canine, and human) previously described in the literature.

## Materials and Methods

### Ethics of Experimentation

The samples were obtained from two trained dolphin males housed at the Oceanogràfic de Valencia following the Animal Care Protocol and policies of the aquarium. The animals were conditioned through positive reinforcement to participate in many different medical behaviors, including semen collection to provide basic husbandry care. Semen samples were obtained through training following the same approach as previously published (Sánchez-Calabuig et al., 2017). Furthermore, the regulations and policies of EU legislation, Directive 2010/63/EU^1^, were observed.

### Animals

Semen samples were obtained from two adult males, who have lived in the same dolphin group since 2003. The estimated ages of both animals were 32 years (Male 1) and 27 years (Male 2) at the moment of the study. Both males live together with a stable social group in an outdoor pool that contains salty water at a temperature of 18–26°C. Both males successfully had previously sired several offspring. Their diet is based on frozen-thawed whole fish (herring*-Clupea harengus*, capelin*-Mallotus villosus*, blue whiting-*Micromesistius poutassou*, mackerel*-Scomber scombrus*, smelt*-Osmeridae*, squid*-Loligo* sp. and European sprat*-Sprattus sprattus*).

### Semen Collection

Two ejaculates from each male were obtained by favoring extraction in the ventrum in an above water level position. The animals always collaborated with the trainers, who, through acoustic and tactile signals, favored extraction of the penis. The stimulation, which lasted a few seconds, ended in ejaculation. Sample was collected in a sterile LLDPE (linear low-density polyethylene) plastic bag (Sánchez-Calabuig et al., 2017). After collection, the following parameters were evaluated for each semen sample (as previously described by Ruiz-Díaz et al., 2020): pH, concentration (number of sperm/ml), total (%) and progressive motility (%) (evaluated by CASA system; 25 frames per second, and at least five random fields per sample), and viability (%) (200 spermatozoa per sample) (Table 1). All the samples were collected within 1 month (the days between sample extractions were 6 and 30 for Male 1 and Male 2, respectively).

**TABLE 1:** Seminal parameters of bottlenose dolphin males (*Tursiops truncatus*) used for proteomic analysis.

**Parameters**	**Male 1**			**Male 2**			**Mean^2^**
	**S1**	**S2**	**Mean^1^**	**S1**	**S2**	**Mean^1^**	
Concentration (×10^6^ sperm/ml)	570.0	780.0	675.0 ± 148.5	200.0	770.0	485.0 ± 403.1	580.0 ± 289.2
pH	8.0	7.0	7.5 ± 0.7	7.5	7.0	7.2 ± 0.4	7.4 ± 0.5
Viability (%)	95.0	92.0	93.5 ± 1.1	88.0	90.0	89.0 ± 0.7	91.2 ± 1.5
Total motility (%)	69.0	77.0	73.0 ± 2.8	88.0	98.0	93.0 ± 3.5	83.0 ± 6.3
Progressive motility (%)	36.0	35.0	35.5 ± 0.4	24.0	29.0	26.5 ± 1.8	31.0 ± 2.8

*S1 and S2 indicate sample 1 and sample 2, respectively.*

*^1^Represents the mean of S1 and S2 for each male.*

*^2^Represents the mean including the samples (S1 and S2) from both males. Mean data for concentration and pH are expressed as mean ± SD (standard deviation). Mean data for viability, total motility and progressive motility are expressed as mean ± SEM (standard error of the mean).*

### Protein Extraction

**FIGURE 1:**
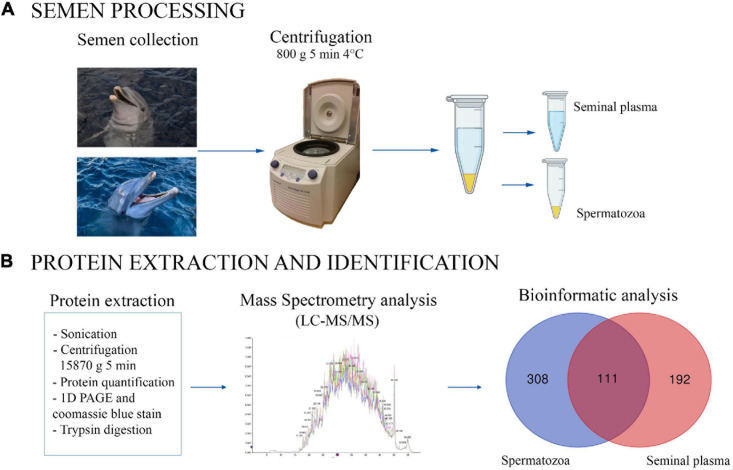
Graphical representation of experimental process for bottlenose dolphin semen processing **(A)** and protein extraction and identification **(B)**. The Venn diagram shows the proteins found to be common (or not) to seminal plasma and spermatozoa.

The proteomic analysis was performed in the proteomics facility of SCSIE, University of Valencia which forms part of ProteoRed, PRB2-ISCIII, supported by grant PT13/0001. Semen samples were centrifugated at 800 × *g* for 5 min at 4°C to split the sample into two fractions (spermatozoa and seminal plasma). The fraction containing spermatozoa was washed twice (800 × *g* for 5 min, 4°C) in phosphate-buffered saline (PBS, Sigma-Aldrich^®^, Madrid, Spain) and the pellet was re-suspended in lysis buffer (50 mM Tris–HCl pH 8, 10 mM DTT and 2% SDS). The mixture was sonicated for 5 min, centrifuged 5 min at 15,870 × *g* and the resulting supernatant was transferred to a fresh microfuge tube (Figure 1). The seminal plasma was centrifuged twice (800 × *g* for 5 min, 4°C) to remove any remaining spermatozoa (microscopically verified).

The protein concentration obtained from both, spermatozoa and seminal plasma samples, was determined by Protein Quantification Assay Kit (Macherey-Nagel, Düren, Germany) according to the manufacturer’s instructions. Twenty micrograms of protein were adjusted to a 40 μl final volume with Laemmli Sample buffer (Bio-Rad, Hercules, CA, United States) with 2–5% β-mercaptoethanol.

#### 1D PAGE

The sample was denatured at 95°C for 5 min and then loaded into a 1D PAGE. Electrophoresis was performed using a 12% precast gel (Bio-Rad, Hercules, CA, United States) at 200 V for 5 min. Then, the gel was fixed with 40% ethanol/10% acetic acid for 1 h, stained with colloidal Coomassie (Bio-Rad, Hercules, CA, United States) for 1 h and scanned by Image Scanner (GE Healthcare).

#### In Gel Protein Digestion

The gel slide was digested with sequencing grade trypsin (Promega, Madison, WI, United States) as described elsewhere (Shevchenko et al., 1996). Briefly, isolated proteins were digested with trypsin (500 ng) at 37°C overnight. The digestion was quenched with 10% trifluoroacetic acid (TFA) at final concentration of 1%. And, after double extraction with neat acetonitrile (ACN), the peptide mixtures were dried in a rotary evaporator and resuspended with 20 μl of 2% ACN, 0.1% TFA.

#### Mass Spectrometry Analysis and Protein Identification

For liquid chromatography and tandem mass spectrometry (LC–MS/MS), 3 μl of a peptide mixture sample was loaded onto a trap column (3 μm C18-CL, 350 μm × 0.5 mm; Eksigent, CA, United States) and desalted with 0.1% TFA at 5 μl/min for 5 min. The peptides were then loaded onto an analytical column (3 μm C18-CL 120 Å, 0.075 × 150 mm; Eksigent, CA, United States) equilibrated with 5% ACN and 0.1% formic acid (FA). Elution was carried out with a linear gradient of 5–40% B in A for 45 min (A: 0.1% FA; B: ACN, 0.1% FA) at a flow rate of 300 nl/min. Peptides were analyzed in a nanoESIqQTOF mass spectrometer (6600plus TripleTOF, ABSciex, MA, United States).

Samples were ionized in a Source Type: Optiflow < 1 μl Nanoapplying 3.0 kV to the spray emitter at 200°C. Analysis was carried out in data-dependent mode. Survey MS1 scans were acquired from 350 to 1,400 m/z for 250 ms. The quadrupole resolution was set to “LOW” for MS2 experiments, which were acquired at 100–1,500 m/z for 25 ms in “high sensitivity” mode. The following switch criteria were used: charge 2+ to 4+; minimum intensity, 250 counts per second (cps). Up to 100 ions were selected for fragmentation after each survey scan. Dynamic exclusion was set to 15 s. The system sensitivity was controlled by analyzing 0.5 μg of K562 trypsin digestion (Sciex). Two thousand, eight hundred and seventy proteins were identified (FDR < 1%) in these conditions in a 45 min gradient.

Data were processed with ProteinPilot v. 5.0 (AB Sciex, Framingham, MA, United States), using the default parameters to generate a peak list directly from 6600 plus TripleTofwiff files. The Paragon algorithm (Shilov et al., 2007) of ProteinPilot v. 5.0 was used to search the Uniprot *Delphinidae* database (v 02.2020; 3513001 proteins in database) with the following parameters: Trypsin enzyme specificity, taxonomy non-restricted, and the search effort was set to rapid.

### Protein Identification

#### Protein Orthologs Identification

Uncharacterized proteins with no properly assigned gene symbol accounted for 15–25% of the proteins within each sample. To identify a direct ortholog for each uncharacterized protein, the proteins were filtered with a custom script written in Python (Supplementary File 1) making use of libraries Bio.Blast (Cock et al., 2009) SeqIO, StrinGIO and pandas. In essence, the algorythm performed parallel queries with the uncharacterized proteins against a database made with “makeblastdb” from a UniProt fasta database made by merging the Swissprot curated database with the *T. truncatus* TrEMBL dataset (release 2020_02). Candidate orthologs were then ranked from maximum to minimum sequence identity and those with a sequence identity within 5% of the maximum were selected. The results were then manually curated, reducing the uncharacterized proteins to 0.2–1% of the proteins within each sample. In some cases, it was not possible to distinguish the direct ortholog within the candidates (the case with several keratins) and thus the proteins where omitted from the biological annotation.

#### Biological Annotation

For the biological annotation, only the proteins identified in all the samples (seminal plasma or spermatozoa) were analyzed. A list with the gene symbols corresponding to each protein was submitted to DAVID [v6.8 (Huang et al., 2009)]. Because of the performance querying *versus* the *T. truncatus* database was very poor, the queries were made with the *Homo sapiens* database, as it is the best annotated organism. Unmapped IDs were checked for synonyms in GeneCard in order to avoid losing information by selecting those identifiers present in DAVID (which were not always the HGNC ones). All the Gene Ontology terms with a FDR < 0.01 were selected and classified into the three categories “biological process,” “cellular component,” and “molecular process.”

The mass spectrometry proteomics data have been deposited to the ProteomeXchange Consortium via the PRIDE (Perez-Riverol et al., 2019) partner repository with the dataset identifier PXD024588.

## Results

A total of 722 different proteins in seminal plasma and spermatozoa were identified in each of the four samples analyzed): 419 proteins in spermatozoa, and 303 in seminal plasma, of which 111 proteins were common to both, spermatozoa and seminal plasma (Figure 1). The total list of identified proteins for each analyzed sample (two per male) is shown in Supplementary File 2 (sperm proteins) and Supplementary File 3 (seminal plasma proteins). Moreover, the Supplementary File 4 shows the list of the common proteins between spermatozoa and seminal plasma.

**FIGURE 2:**
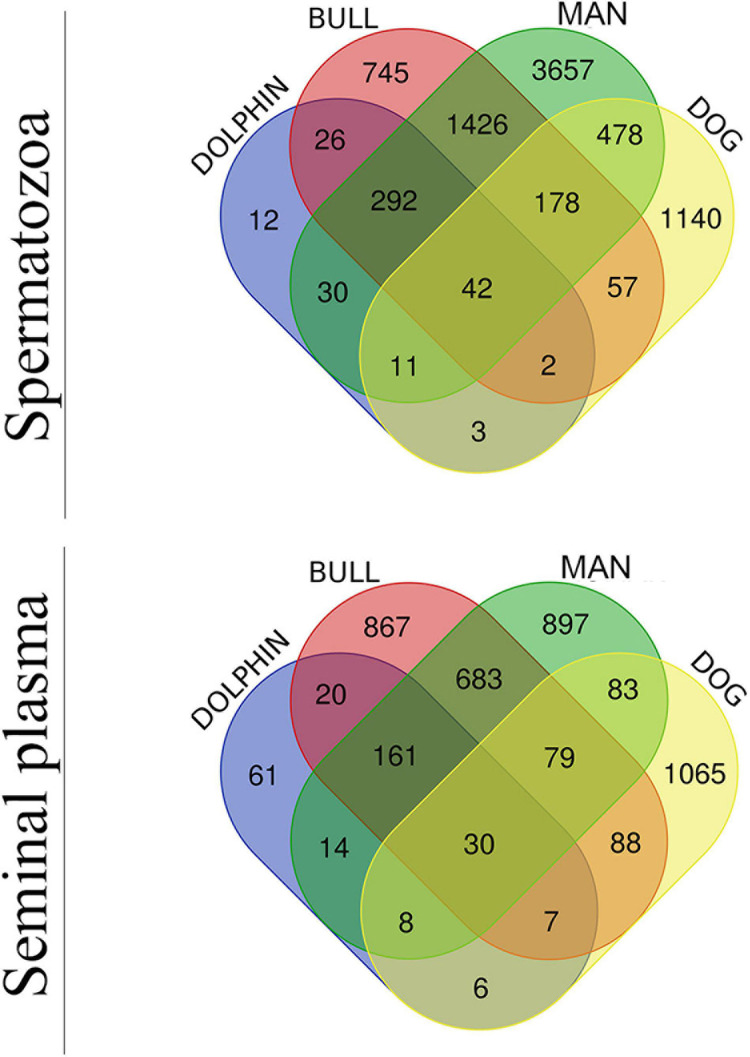
Venn diagram of sperm (upper diagram) and seminal plasma (lower diagram) proteomes of man, bull, dog and dolphin.

Since bovids and cetaceans are evolutionarily related (Vislobokova, 2013), belonging to the order Cetartiodactyla, a bovine dataset (Ramesha et al., 2020) was compared with dolphin. In addition, human as a biological model (Batruch et al., 2011; Baker et al., 2013; Amaral et al., 2014) and dogs (canids) (Aquino-Cortez et al., 2017; Araujo et al., 2020), because their only accessory sex gland is the prostate, as in dolphins, were also included in the comparison. The four species were therefore retrieved to evaluate the proteome profile of bottlenose dolphin spermatozoa and seminal plasma, resulting in the Venn diagram of Figure 2. The analysis revealed that 42 were identified in the spermatozoa of all four species, and 30 in the seminal plasma of the same. The number of co-present sperm proteins between animals were the following: (i) bull and dolphin, 362; (ii) man and dolphin, 375; and (iii) dog and dolphin, 58. The number of co-present seminal proteins were: (i) bull and dolphin, 218; (ii) man and dolphin, 213; and (iii) dog and dolphin, 51. These proteins are listed in Supplementary File 5 (sperm) and Supplementary File 6 (seminal plasma).

### Protein Profile of Bottlenose Dolphin Spermatozoa

As already mentioned, a total of 419 proteins were present in the spermatozoa of the four samples analyzed (two from each male). A complete protein list (ordered by the number of peptides) for the spermatozoa accompanied by the protein name, String ID dolphin, peptides, mean protein score (±SD), preferred name and annotation (*H. sapiens*) is presented in Supplementary File 7. A detailed search was carried out in the STRING database v 11.0^2^ to obtain a description of the functions related with each of the proteins. The following cases were observed: (i) Proteins described for *T. truncatus* (269 proteins, 63.9%); (ii) Proteins described for *H. sapiens* (416 proteins, 99.3%); and (iii) Proteins not described in either of the above (3 proteins, 0.7%). Of the proteins belonging to the third case, one is described for *Mus musculus* (ADAM5), one in *Pan paniscus* (LRRC37A5P) and the other one has no description for any species (ATP6V1FNB).

**FIGURE 3:**
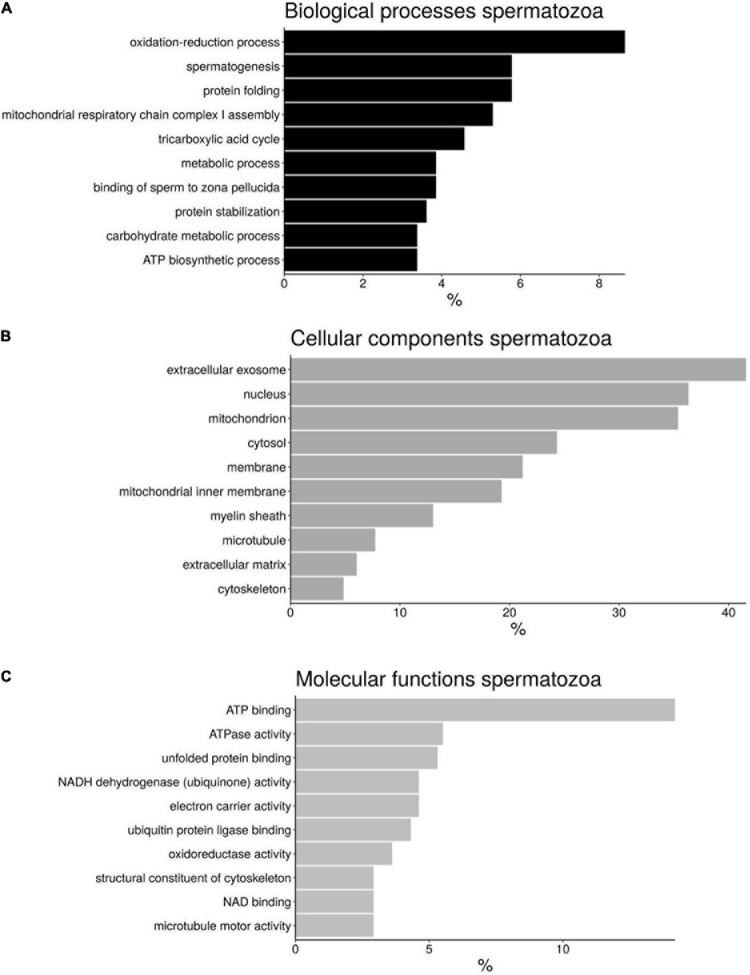
Bar chart representing the gene ontology annotations of protein identified in bottlenose dolphin spermatozoa according to biological processes **(A)**, cellular components **(B)**, and molecular functions **(C)**.

A functional annotation of the proteins from *H. sapiens* sperm was peformed using DAVID software and divided into different categories: “Biological processes” (BP), “Cellular components” (CC), and “Molecular functions” (MF) (Figure 3 and Supplementary File 8). Although there are different categories for each of the divisions made, the most abundant were those involved in oxidation-reduction processes (8.4%), spermatogenesis (6%), and protein folding (6%) in the case of BP (Figure 3A); extracellular exosome (41.6%), nucleus (36.3%), and mitochondrion (35.3%) in the case of CC (Figure 3B); and ATPbinding proteins (14.2%), ATPase activity (5.5%), and unfolded protein binding (5.3%) for MF (Figure 3C).

Of the total of 419 proteins identified in the spermatozoa, 39 are linked to sperm function categories (Table 2) such as cilia/flagela (17 proteins, 43.6%), sperm motility (10 proteins, 25.6%), capacitation and acrosome reaction (4 proteins, 10.3%), sperm-egg fusion (4 proteins, 10.3%), spermatogenesis and fertilization (2 proteins, 5.1%), and other functions (2 proteins, 5.1%). Not all 39 proteins involved in sperm functions are identified and/or described in the STRING program for dolphins, so we divided them into three different categories: (1) Proteins described for *T. truncatus* (27 proteins, 69.2%); (2) Proteins described for *H. sapiens* (35 proteins, 89.7%); and (3) Proteins described for *M. musculus* (1 protein, 2.6%). Twenty-four proteins were common to both categories (1 and 2).

**TABLE 2:** Proteins identified in dolphin spermatozoa linked to sperm function categories.

**Function**	**Gene name**	**Protein**
**Spermatogenesis and fertilization**	NME5 (1,2)	NME/NM23 family member 5 (NME5)

	SEPT12 (1,2)	Septin 12 (SEPT12)
**Sperm–egg fusion**	CD46 (2)	CD46 molecule(CD46)
	PKD2L2 (2)	Polycystin 2 like 2, transient receptor potential cation channel (PKD2L2)
	SPACA1 (1,2)	Sperm acrosome-associated 1 (SPACA1)
	ADAM5 (3)	ADAM metallo peptidase domain 5 (ADAM5)

**Capacitation and acrosome reaction**	ACRBP (1,2)	Acrosin binding protein (ACRBP)
	AKAP3 (1,2)	A-kinase anchoring protein 3 (AKAP3)
	CABYR (1,2)	Calcium binding tyrosine phosphorylation regulated (CABYR)
	DLD (1,2)	Dihydrolipoamide dehydrogenase (DLD)

**Sperm motility**	DNAH1 (1,2)	Dyneinaxonemal heavy chain 1 (DNAH1)
	DNAH10 (1,2)	Dyneinaxonemal heavy chain 10 (DNAH10)
	DNAH12 (1,2)	Dyneinaxonemal heavy chain 12 (DNAH12)
	DNAH17 (1,2)	Dyneinaxonemal heavy chain 17 (DNAH17)
	DNAH2 (1,2)	Dyneinaxonemal heavy chain 2 (DNAH2)
	DNAH3 (1,2)	Dyneinaxonemal heavy chain 3 (DNAH3)
	DNAH8 (1,2)	Dyneinaxonemal heavy chain 8 (DNAH8)
	EFHB (1,2)	EF-hand domain family member B (EFHB)
	ROPN1L (1,2)	Rhophilin-associated tail protein 1 like (ROPN1L)
	SLC25A31 (1,2)	Solute carrier family 25 member 31 (SLC25A31)

**Cilia and flagela**	CALM1 (1,2)	Calmodulin 1 (CALM1)
	CFAP20 (2)	Cilia- and flagella-associated protein 20 (CFAP20)
	CFAP43 (2)	Cilia- and flagella-associated protein 43 (CFAP43)
	CFAP52 (2)	Cilia- and flagella-associated protein 52 (CFAP52)
	CFAP58 (2)	Cilia- and flagella-associated protein 58 (CFAP58)
	CFAP61 (2)	Cilia- and flagella-associated protein 61 (CFAP61)
	DNALI1 (1,2)	Dyneinaxonemal light intermediate chain 1 (DNALI1)
	EFHC1 (1,2)	EF-hand domain containing 1 (EFHC1)
	ODF1 (1)	Outer dense fiber of sperm tails 1 (ODF1)
	ODF2 (1)	Outer dense fiber of sperm tails 2 (ODF2)
	ODF3 (1)	Outer dense fiber of sperm tails 3 (ODF3)
	PRKACB (2)	Protein kinase cAMP-activated catalytic subunit beta (PRKACB)
	RSPH9 (1,2)	Radial spoke head 9 homolog (RSPH9)
	SAXO1 (2)	Stabilizer of axonemal microtubules 1 (SAXO1)
	TBC1D21 (1,2)	TBC1 domain family member 21 (TBC1D21)
	TUBB (2)	Tubulin beta class I (TUBB)
	TUBB4B (2)	Tubulin beta 4B class IVb (TUBB4B)

**Gonadal development**	BANF1 (1,2)	Barrier to autointegration factor 1 (BANF1)

**Testis**	TCTE1 (1,2)	T-complex-associated-testis-expressed 1; Dynein regulatory complex (TCTE1)

*The numbers in the “gene name” column refer to the source organism in which the proteins are described according to the STRING database: (1) described in Tursiops truncatus; (2) described in Homo sapiens; and (3) described in Mus musculus.*

### Protein Profile of Bottlenose Dolphin Seminal Plasma

A total of 303 proteins were identified in the seminal plasma of the four samples obtained, two from each male. A complete protein list (ordered by the number of peptides) for the seminal plasma with protein name, String ID dolphin, peptides, mean protein score (±SD), preferred name and annotation (*H. sapiens*) is presented in Supplementary File 9. A detailed search was carried out in the STRING database v 11.0 (see text foot note 2) to obtain a description of the functions related to each of the proteins, finding the following: (i) Proteins described for *T. truncatus* (201 proteins, 66.1%); (ii) Proteins described for *H. sapiens* (298 proteins, 98.0%); (iii) Proteins not described in any of above (4 proteins, 1.3%). Of the proteins belonging to the third case, all are described for *M. musculus* (ADAM1, ADAM5, DNAJB3 y MUC19), an animal with poliandric reproduction and sperm competition.

**FIGURE 4:**
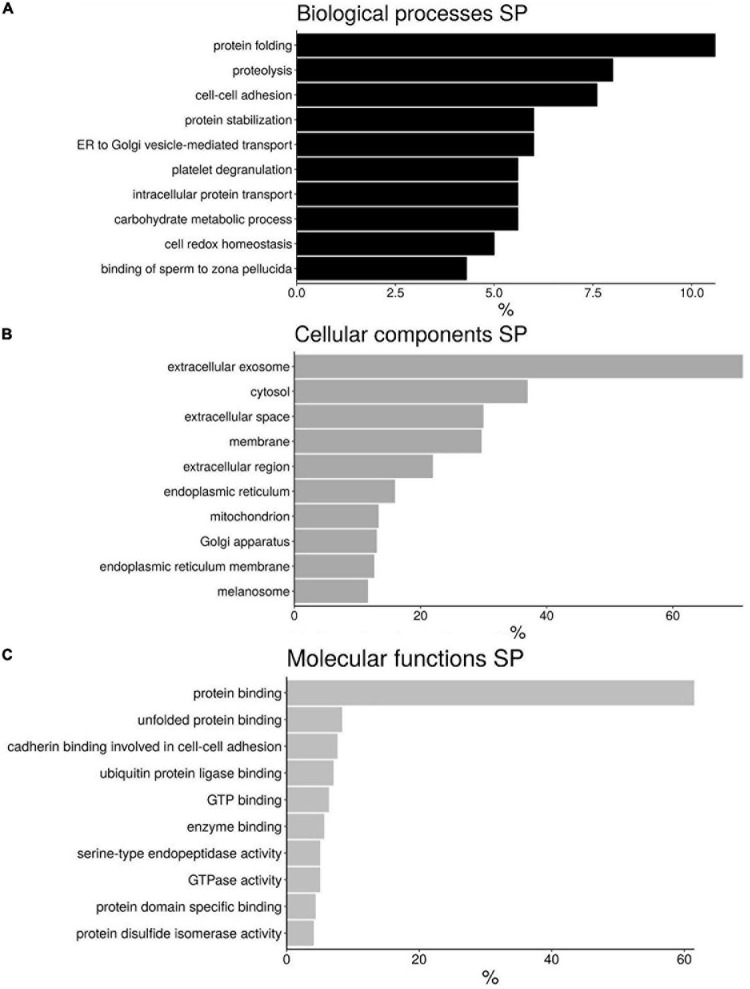
Bar chart representing the gene ontology annotations of protein identified in bottlenose dolphin seminal plasma (SP) according to biological processes **(A)**, cellular components **(B)**, and molecular functions **(C)**.

The proteins present in seminal plasma described in *H. sapiens* were distributed into 3 categories according DAVID software: “Biological process” (BP), “Cellular components” (CC), and “Molecular Function” (MF) (Figure 4 and Supplementary File 8). Although there are different categories for each of the divisions made the most abundant are those involved in protein folding (10.6%), proteolysis (8%), and cell–cell adhesion (7.6%) for BP (Figure 4A); extracellular exosome (71.1%), cytosol (36.9%), and extracellular space (29.9%) for CC (Figure 4B); and protein binding (61.5%), unfolded protein binding (8.3%) and cadherin binding involved in cell-cell adhesion (7.6%) for MF (Figure 4C).

Of the 303 proteins identified in seminal plasma, 31 are involved in reproductive processes (Table 3) such as adhesion with zona pellucida (8 proteins, 25.8%), spermatogenesis (6 proteins, 19.3%), sperm motility process (4 proteins, 12.9%), the formation of microtubules involved in flagellar development (4 proteins, 12.9%), sperm maturation/capacitation (4 proteins, 12.9%), epididymal proteins (2 proteins, 6.5%), muellerian inhibitor (1 protein, 3.2%), oocyte maturation (1 protein, 3.2%), and testis protein (1 protein, 3.2%). Not all the 31 reproductive proteins are identified and/or described in the STRING program for dolphins, so we divided them into three different categories: (1) Those described in *T. truncatus* (29 proteins, 93.5%); (2) Those described in *H. sapiens* (17 proteins, 54.8%); and (3) Those described in *M. musculus* (2 proteins, 6.7%). All the proteins described in *H. sapiens* are also present in *T. truncatus.*

**TABLE 3:** Proteins identified in dolphin seminal plasma linked to sperm function categories.

**Function**	**Gene name**	**Protein**
**Sperm motility (energy)**		
	AK1 (1)	Adenylate kinase 1 (AK1)
	APOA1 (1,2)	Apolipoprotein A1 (APOA1)
	CKMT1 (1)	Creatine kinase mitochondrial 1A (CKMT1A)
	ROPN1 (1)	Rhophilin-associated tail protein 1 (ROPN1)

**Sperm maturation/capacitation**		
	ADAM1 (3)	ADAM metallo peptidase domain 1 (ADAM1)
	CST11 (1,2)	Cystatin-11 (CST11)
	AKAP3 (1,2)	A-kinase anchoring protein 3 (AKAP3)
	ROPN1L (1,2)	Rhophilin-associated tail protein 1 like (ROPN1L)

**Sperm adhesion zona pellucida**		
	ADAM2 (1)	ADAM metallo peptidase domain 2 (ADAM2)
	ADAM5 (3)	ADAM metallo peptidase domain 5 (ADAM5)
	CD9 (1,2)	CD9 molecule (CD9)
	CLGN (1,2)	Calmegin (CLGN)
	FETUB (1)	Fetuin B (FETUB)
	MFGE8 (1)	Milk fat globule-EGF factor 8 protein (MFGE8)
	PRSS37 (1,2)	Protease, serine 37 (PRSS37)
	TEX101 (1,2)	Testis expressed 101 (TEX101)

**Epididymal**		
	LCN6 (1,2)	Lipocalin 6 (LCN6)
	NPC2 (1)	NPC intracellular cholesterol transporter 2 (NPC2)

**Muellerian inhibitor**		
	AMH (1,2)	Anti-Muellerian hormone (AMH)

**Oocyte maturation**		
	CALR (1,2)	Calreticulin (CALR)

**Spermatogenesis**		
	BSG (1,2)	Basigin (Ok bloodgroup) (BSG)
	NAP1L4 (1,2)	Nucleosome assembly protein 1 like 4 (NAP1L4)
	PRKACB (1)	Protein kinase cAMP-activated catalytic subunit beta (PRKACB)
	RNASE10 (1)	Ribonuclease A (RNASE10)
	SHCBP1L (1,2)	SHC binding and spindle-associated 1 like (SHCBP1L)
	SPAG4 (1,2)	Sperm-associated antigen 4 (SPAG4)

**Testis**		
	PDILT (1,2)	Protein disulfide isomerase like, Testis expressed (PDILT)

**Microtubules: cilia and flagella**		
	CFAP36 (1)	Cilia- and flagella-associated protein 36 (CFAP36)
	PCMT1 (1,2)	Protein-l-isoaspartate (d-aspartate) *O*-methyl transferase (PCMT1)
	TUBB (1)	Tubulin beta class I (TUBB)
	TUBB4B (1)	Tubulin beta 4B class IVb (TUBB4B)

*The numbers in the “gene name” column refer to the source organism where the proteins are described according to the STRING database: (1) described in *Tursiops truncatus;* (2) described in *Homo sapiens*; and (3) described in *Mus musculus.**

### Protein–Protein Interaction Networks

**FIGURE 5:**
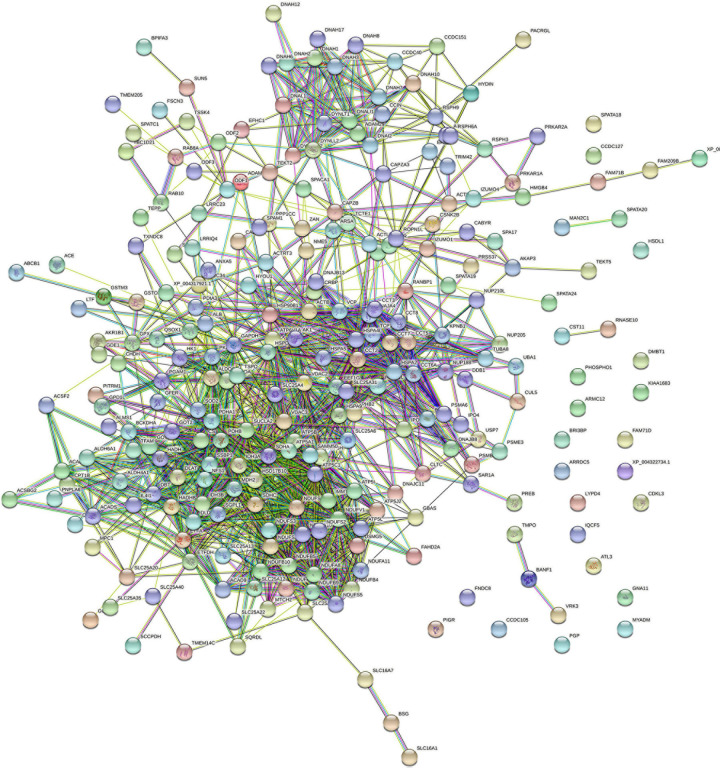
STRING protein–protein interaction network showing the interactions of the spermatozoa proteins identified in bottlenose dolphin.

**FIGURE 6:**
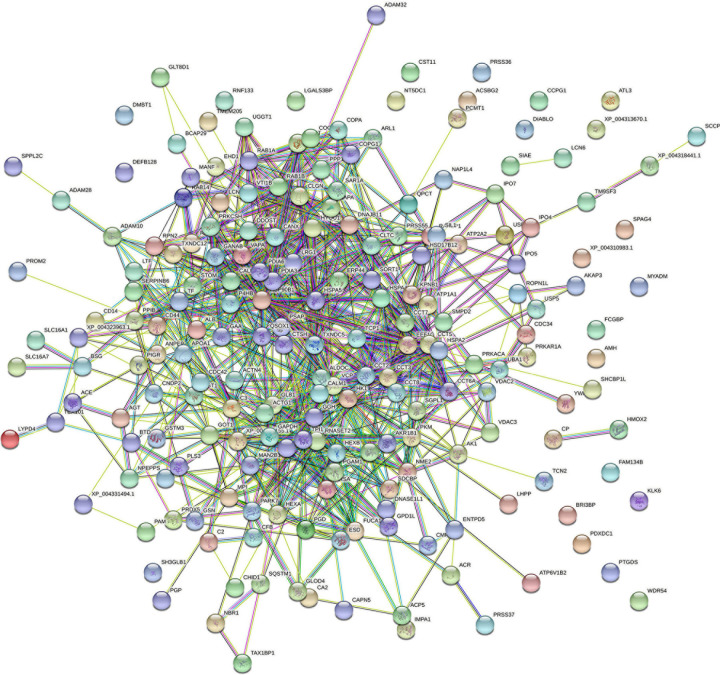
STRING protein–protein interaction network showing the interactions of the seminal plasma proteins identified in bottlenose dolphin.

All proteins were searched using STRING software. A total of 266 nodes and 1,452 edges were identified in *T. truncatus* spermatozoa (Figure 5), as well as 201 nodes and 804 edges in seminal plasma (Figure 6) for the protein–protein interaction (PPI) networks. Network nodes represent proteins and the line color indicates the type of interaction evident, light blue color for known interactions from curated databases, and pink color for interactions experimentally determined. The Predicted Interactions are represented by different colors: green for gene neighborhood, red for gene fusions and dark blue for gene co-occurrence. Other relations between proteins are identified with light green for text mining, black for co-expression and blue for protein homology. Query proteins are represented as colored nodes and the second shell of interactors are represented as white nodes. Empty nodes represent proteins of unknown 3D structure and filled nodes represent predicted or known structures.

## Discussion

Dolphins are one of the most studied marine mammals, mainly due to their easy maintenance in *ex situ* conditions compared to other cetaceans. Although dolphins can reproduce in captivity, the main problem is the inbreeding that occurs in small groups of animals. For this reason, their reproductive characteristics and subsequent improvements in ARTs are essential in these animals to ensure genetic spread and conservation. In this respect, studies of the semen protein composition has determined the possibility of using sperm freezing in different species (Soleilhavoup et al., 2014; Kasimanickam et al., 2019; Gaitskell-Phillips et al., 2021; Martínez-Fresneda et al., 2021), the identification of presumptive fertility biomarkers (Dacheux et al., 2012; Li et al., 2016; Rahman et al., 2017; Druart and de Graaf, 2018; Pérez-Patiño et al., 2018; Druart et al., 2019) or sperm traits (Intasqui et al., 2016; Bezerra et al., 2019; De Lazari et al., 2019). The present study describes the proteins in spermatozoa and seminal plasma of bottlenose dolphin. To the best of our knowledge, this study represents the first description of the semen proteome in any cetacean. A total of 722 proteins (common to the 4 samples analyzed) were described for sperm (419) and seminal plasma (303).

### Proteins Identified in the Spermatozoa of Bottlenose Dolphin

The five most abundant proteins found in dolphin spermatozoa (based on the number of peptides identified) were the following: AKAP3, ODF2, TUBB, GSTM3, and ROPN1. Four of these proteins (AKAP3, ODF2, TUBB, and GSTM3) are also in the top five of highly abundant proteins in the spermatozoa of buffalo (Fu et al., 2019). AKAP3 protein was the most abundant sperm protein according to our data. This protein is not only essential for the formation of the subcellular structure of the sperm flagellum, sperm motility and male fertility in mice, but also for sperm capacitation. As is known, sperm capacitation is a series of biochemical and physiological changes that mammalian sperm must undergo to become fertile (Gervasi and Visconti, 2016). AKAP3 null mice sperm has low sperm motility, leading to sperm morphology abnormalities, the displacement of PKA subunits and misregulated PKA activity, a key factor during sperm capacitation, and in male sterility (Xu et al., 2020). Moreover, other proteins identified in our study, such as CABYR and ROPN1, interact with AKAP3 (Carr et al., 2001; Li et al., 2011). CABYR protein is a fibrous sheath calcium-binding tyrosine phosphorylation-regulated protein (Li et al., 2007), which associates with AKAP3 in human spermatozoa (Li et al., 2011). CABYR is located in the principal piece of the sperm flagellum (Li et al., 2007; Zhang et al., 2016) and it is involved in calcium-binding when phosphorylated during capacitation (Naaby-Hansen et al., 2002). Moreover, this protein is expressed in the oviduct of several species (Hanlon Newell et al., 2008; Martinez et al., 2020), where its expression increases in response to sperm entry. ODF2 is the second most abundant protein found in the spermatozoa of bottlenose dolphin. In mammalian sperm, the flagellum presents complex accessory structures surrounding the central axoneme, the outer dense fibers (ODFs) being part of these structures (Zhao et al., 2018). Dolphin sperm is not an exception, and ODFs are almost of similar form and size as in most other mammals (van der Horst et al., 2018). ODFs play a role in the protection of the sperm tail against shear forces during epididymal transport and ejaculation (Baltz et al., 1990). More than 14 polypeptides from mammalian ODFs have been identified, including four major proteins (ODF1, ODF2, ODF3, and ODF4) (reviewed by Zhao et al., 2018). Our analysis identified ODF1, ODF2, and ODF3 in dolphin sperm, the most abundant being ODF2 protein. The disruption of ODF2 expression in mice reduced sperm motility, and is compatible with asthenozoospermia characteristics (Zhao et al., 2018). Moreover, ODF2 is indispensable for the neck midpiece connection, which is composed of a centrosome-derived component and a flagellar component (Ito et al., 2019). Tubulins are a family of proteins in which α- and β-tubulin are the major components of microtubules in spermatozoa (Kierszenbaum, 2002). TUBB and TUBB4B proteins are two of the tubulin proteins previously be found in the sperm of our analyzed dolphin samples. These proteins are important in the development of cilia and flagellum, but there is nothing in the bibliography which explains in detail the relation between this fact and the presence/absence of this group as fertility biomarkers, although TUBB protein exhibits clear differences in expression according to porcine litter size (Kwon et al., 2015b). GSTM3 is another protein situated among the five most abundant sperm proteins identified in our study. GSTM3 is a detoxification protein, which has been reported to play a key role in oocyte binding (Gopalakrishnan et al., 1998; Petit et al., 2013) and their interaction with the zona pellucida (Petit et al., 2013). Moreover, sperm GSTM3 has been proposed as a quality (Llavanera et al., 2020b), fertility (Kwon et al., 2015a) and cryotolerance (Llavanera et al., 2019) biomarker for pig sperm. Our data also report the presence of this protein in the seminal plasma of dolphins. Recently, it has been demonstrated that low concentration of GSTM3 in pig seminal plasma is related to an increased percentage of sperm abnormalities (Llavanera et al., 2020a). ROPN1 protein is found in sperm flagellum as part of the fibrous sheath, specifically located in the principal piece and end piece of the flagellum (Fujita et al., 2000; Chen et al., 2011). The expression level of ROPN1 was found to be significantly lower in asthenozoospermic men than in normozoospermic suggesting its involvement in sperm motility (Chen et al., 2011). When mice lacking ROPN1 were analyzed, the sperm exhibited altered motility, the same males being subfertile, and producting fewer and smaller litters (Fiedler et al., 2012). Indeed, a positive correlation between motility (progressive and total motile sperm number) and ROPN1/CABYR gene expression has been observed (Pelloni et al., 2018).

One interesting sperm protein identified in our data was IZUMO1. Sperm-egg fusion is accomplished through the interaction of a specific membrane proteins in sperm, IZUMO1 (Inoue et al., 2005), and oocyte, JUNO (Bianchi et al., 2014). During acrosome reaction, IZUMO1 relocates from the anterior head of the sperm to the site where fusion takes place and which was the first site to be shown as critical for gamete fusion (Inoue et al., 2005, 2011). Thus, our data suggest that gamete fusion is probably mediated by the same mechanism as that described in mouse.

### Proteins Identified in the Seminal Plasma of Bottlenose Dolphin

As mentioned above, the seminal plasma of dolphins comes from the testis, epididymis and the prostate, the only accessory sex gland in this species (Harrison et al., 1969; Rommel et al., 2007; Suárez-Santana et al., 2020). Seminal plasma is not simply a transport fluid for sperm but also modulates the female genital tract environment (Bromfield, 2014). It also protects sperm during their journey toward the oocyte (Kawano et al., 2014; Luongo et al., 2019) and interacts with other reproductive fluids modifying the sperm proteome (Luongo et al., 2020). The five most abundant proteins (based on the number of peptides identified) represented in seminal plasma of dolphin were CST11, LTF, ALB, HSP90B1, and PIGR. Of these CST11 (Cystatin11) was seen to be the most abundant protein in the seminal plama of dolphin (and was also detected in the sperm). CST11 belongs to the cystatin type 2 family of cysteine protease inhibitor and exhibits antimicrobial activity *in vitro* (Magister and Kos, 2013), suggesting that cystatins defend the male reproductive tract against invading pathogens (Hamil et al., 2002; Fan et al., 2014). This protein has been detected throughout the epididymis (particularly in the initial segment) and in ejaculated human sperm (Hamil et al., 2002). LTF is a glycoprotein with antioxidant and antibacterial activities that is synthesized in the epididymis in mammals (Pearl and Roser, 2008) and has been detected in the prostate and seminal vesicles in humans (Wichmann et al., 1989). It has been reported that the treatment of asthenoteratozoospermic males with LTF, combined with other natural antioxidants, significantly improves the motility of sperm cells (Piomboni et al., 2008). Moreover, a positive correlation between seminal plasma LFT concentration and sperm density was established in dogs (Kikuchi et al., 2003a) and horses (Kikuchi et al., 2003b). The addition of LTF to freezing sperm extender protected stallion sperm, increasing the percentage of sperm with functional membranes and decreased lipid oxidizing agents (Martins et al., 2018). ALB protein is positively related with sperm concentration, total sperm count and the percentage of morphologically normal spermatozoa, but negatively related with semen volume in humans (Elzanaty et al., 2007). In a study developed in Holstein bulls, high concentrations of this protein correlated with animals whose semen was classified as highly fertile (Kasimanickam et al., 2019), so this protein could be used as a biomarker of fertility. HSP90B1 is a molecular chaperone member of the heat shock protein 90 (HSP90) family. This protein is involved in the immune response (Graustein et al., 2018) in the suppression of cell apoptosis and autophagy (Sun et al., 2015). This function suggests a putative role for HSP90B1 once it is in the female genital tract after insemination, since it is known that seminal plasma modulates maternal immunity (Bromfield, 2018). PIGR is involved in the tissue homeostasis pathway (Panner Selvam et al., 2019) and its expression is regulated by cytokines, which show high levels in the seminal plasma of varicocele patients due to an inflammatory response (Zeinali et al., 2017). The presence of PIGR was previously reported in the semen of bulls (Rego et al., 2014) and cats (Mogielnicka-Brzozowska et al., 2020). In humans, PIGR expression was observed in the prostate (Sirigu et al., 1995) which agrees with our results as the prostate is the only accessory sex gland in dolphins.

NPC2 is one of the proteins found in abundance in the seminal plasma of bottlenose dolphin (the sixth most abundant protein). NPC2 has also been described to be among the most abundant secreted proteins in the epididymis in bovine (Belleannée et al., 2011). In the epididymal fluid, NPC2 participates in cholesterol efflux from the spermatozoa during epididymal sperm maturation (Légaré et al., 2006). Furthermore, capacitated NPC2^(–/–)^ mice spermatozoa exhibited defective tyrosine phosphorylation patterns and a reduced ability to fertilize cumulus-oocyte complexes compared with wild-type spermatozoa, supporting the relevance of epididymal NPC2 in male mouse fertility (Busso et al., 2014). Recently, relative levels of two isoforms of NPC2 were found to be higher in the porcine seminal plasma of poor freezability ejaculates than in that with good freezability, suggesting that the NPC2 content may be useful for predict ejaculate freezability (Valencia et al., 2020).

### Proteome Profile of Dolphin Spermatozoa and Seminal Plasma Compared to Other Species (Human, Bovine, and Canine)

Comparative analyses and a Venn diagram were made relating dolphin to other three species: bovine, canine and human. Bovine and porcine species are even-toed ungulates (artiodactyls), and closest living relatives of dolphin (Thewissen et al., 2007; Spaulding et al., 2009). Actually, both species (bovine and delphinus) share 362 of the 419 sperm proteins detected in the analysis (86.4%) and 218 of the 304 detected in seminal plasma protein (71.7%), appointing to the high number of conserved proteins between these animals. Likewise, dolphin sperm are able to attach and penetrate bovine ZP, even triggering the blockage of polyspermy (Sánchez-Calabuig et al., 2015a, 2017). The dog was also compared to dolphin because they both share the fact that of having only one accessory sex gland, the prostate. Dog and dolphin share 51 seminal plasma proteins, only 6 of which are exclusive to these animals (NBR1, BCAP29, KLK6, SQSTM1, SLC16A7, and MUC19). This suggests that, although prostate is the only accessory sex gland common to dogs and dolphins, the protein composition is not well conserved between species, and less than 25% of the seminal plasma proteins are shared. Moreover, as an outgroup, we also included human in the comparative analysis because of the availability proteomic data. The results indicated that 42 sperm proteins and 30 seminal plasma proteins are common to the four species.

FSIP2 is one of the sperm proteins identified as common to all four compared species (Supplementary File 5) (sixth position in the sperm protein list based on the number of identified peptides; Supplementary File 7). FISP2 is one of the main genes involved in the multiple morphological abnormalities of sperm flagella syndrome (Martinez et al., 2018). Moreover, mutations in FSIP2 lead to the absence of A-kinase anchoring protein 4 (AKAP4), a protein also detected in dolphin sperm. DNAH8 has also been found also to be common to the four species analyzed (Supplementary File 5) (tenth position in sperm protein list based on the number of identified peptides; Supplementary File 7). Loss-of-function mutation in DNAH8 is suggested to cause male infertility because of the multiple morphological abnormalities of sperm flagella syndrome, DNAH8 being essential for sperm flagellum formation (Yang et al., 2020). There are 12 sperm proteins that have only been identified in dolphin: LRRC37A5P, CFAP61, HOGA1, CCDC127, UBB, C6orf58, CST11, DEFB130, HEL-S-80P, ADAM10, ATP6V1FNB, and GCAT.

In the case of seminal plasma, LTF and ALB are present in all the compared species. ENO1, which has been recognized as a candidate for fertility marker in bulls (Park et al., 2019) is another protein conserved in the four species and a good freezability predictor in human (Jiang et al., 2015). It would be interesting to analyze ENO1 in dolphins with different degrees of fertility and as a possible component of an extender during preservation. A total of 61 proteins were exclusively detected in the sperm of dolphin but not in dogs, humans or bulls.

To evaluate whether the most abundant protein found in dolphin are also in the semen of other species (human and bovine) the most abundant protein situated in the first decile of the lists for sperm and seminal plasma of these species were analyzed. In the case of sperm proteins, 16 of them were shared across the three species (DNAH8, ODF2, NDUFS1, HSPA2, TUBB4B, HK1, TEKT5, DLAT, AKAP3, TEKT2, EFHC1, LTF, GSTM3, SDHA, TEKT3, and UQCRC2). Four of them are included in the top ten of the most abundant proteins detected in dolphin sperm (DNAH8, ODF2, AKAP3, and GSTM3). In the case of seminal plasma, 13 proteins were shared between the three species (ACE, QSOX1, PDIA3, P4HB, HYOU1, HSPA5, ALB, GLB1, PIGR, LTF, HSP90B1, HSPA8, and HSP90AA1). Seven of them are included in the top ten of the most abundant proteins detected in the seminal plasma of dolphin (ACE, PDIA3, ALB, PIGR, LTF, HSP90B1, HSPA8, and HSP90AA1).

Protein differences between the species could be a result of different requirements for spermatozoa to interact with the female tract (Druart and de Graaf, 2018). The species here compared (human, bovine, canine, and dolphin) have vaginal semen deposition which can explain a similar protein profile between species for helping with cervical migration. Moreover, cross-species comparison of mammalian seminal plasma proteomes performed in seven species (bovine, sheep, goat, pig, horse, camel, and alpaca) revealed that the phylogenetic proximity between species could be related to the similarity of seminal plasma proteome (Druart et al., 2013).

## Conclusion

In conclusion, this study provides an inventory of 722 proteins present in bottlenose dolphin semen. As demonstrated in most mammalian species, bottlenose dolphin semen proteome includes proteins that are essential to the sperm structure and function. Comparison of this proteome with other physio-pathologies enable the identification of semen markers for reproduction purposes. Moreover, this work opens the door to future research aimed at investigating a new molecular basis of sperm, proteins that are involved in sperm conservation, or those that can be used as biomarkers of sperm quality and fertility.

## Data Availability Statement

The mass spectrometry proteomics data have been deposited to the ProteomeXchange Consortium via the PRIDE partner repository with the dataset identifier PXD024588.

## Ethics Statement

The samples were obtained from two trained dolphin males housed at the Oceanogr fic de Valencia following the Animal Care Protocol and policies of the aquarium. The animals were conditioned through positive reinforcement to participate in many different medical behaviors, including semen collection to provide basic husbandry care. Semen samples were obtained through training following the same approach as previously published (Sánchez-Calabuig et al., 2017). Furthermore, the regulations and policies of EU 143 legislation, Directive 2010/63/EU (https://eur-lex.europa.eu/legal144content/EN/TXT/?uri=CELEX:32010L0063), were observed.

## Author Contributions

MI and FG-V: conceptualization. M-CF-A, LG-B, PC, CL, SA-S, JR-S, EP, SR-D, CB-G, and DG-P: methodology. FG-V: validation and supervision. LG-B: formal analysis and data curation. M-CF-A, LG-B, CL, SA-S, JR-S, EP, SR-D, CB-G, M-JS-C, DG-P, MA, MI, and FG-V: investigation. M-CF-A, CB-G, and DG-P: resources. M-CF-A and FG-V: writing – original draft preparation. M-CF-A, LG-B, PC, CL, SA-S, JR-S, EP, SR-D, CB-G, M-JS-C, DG-P, MA, MI, and FG-V: review and editing. DG-P, MA, and FG-V: funding acquisition. All authors have read and agreed to the published version of the manuscript.

## Conflict of Interest

The authors declare that the research was conducted in the absence of any commercial or financial relationships that could be construed as a potential conflict of interest.

## Footnotes

1 https://eur-lex.europa.eu/legal-content/EN/TXT/?uri=CELEX:32010L0063

2 https://string-db.org/

## Supplementary Material

Supplementary File 1 Python code used to identify the most similar orthologs for dolphin proteins without an official name. The code performs blast queries against a database composed of curated entries from Swissprot plus the Uniprot entries associated with *T. truncatus* (release 2020_02). Candidates for each query are selected within the top 5 matches with an official name, e-value of 0.001 and sequence identity of 60%. If the top match has over 99% identity its name is assigned to the problem protein of dolphin. Otherwise, all candidates within a margin of 5% identity are selected and their names compared. If there is no consensus for the name, the program writes their names and sequence identity and the alignment results are manually checked. If the program does not find any proteins matching the requisites a message is returned: “Check this protein, there are no near orthologs.”

Supplementary File 2 Individual lists of proteins identified in each of the sperm samples analyzed in bottlenose dolphin (*Tursiops truncatus*).

Supplementary File 3 Individual lists of proteins identified in each of the seminal plasma samples analyzed in bottlenose dolphin (*Tursiops truncatus*).

Supplementary File 4 List of common proteins to spermatozoa and seminal plasma in bottlenose dolphin (*Tursiops truncatus*).

Supplementary File 5 List of sperm proteins common (or not) to bull, man, dog, and bottlenose dolphin.

Supplementary File 6 List seminal plasma proteins common (or not) to bull, man, dog, and bottlenose dolphin.

Supplementary File 7 Proteins identified in the sperm (common to all of the analyzed samples) of bottlenose dolphin (*Tursiops truncatus*).

Supplementary File 8 Functional distribution of bottlenose dolphin (*Tursiops truncatus*) spermatozoa and seminal plasma from DAVID Bioinformatics Resources.

Supplementary File 9 Proteins identified in the seminal plasma (common to all of the analyzed samples) of bottlenose dolphin (*Tursiops truncatus*).
